# Therapeutic Touch Has Significant Effects on Mouse Breast Cancer Metastasis and Immune Responses but Not Primary Tumor Size

**DOI:** 10.1155/2015/926565

**Published:** 2015-05-31

**Authors:** Gloria Gronowicz, Eric R. Secor, John R. Flynn, Evan R. Jellison, Liisa T. Kuhn

**Affiliations:** ^1^Department of Surgery, University of Connecticut Health Center, Farmington, CT 06030, USA; ^2^Department of Immunology, University of Connecticut Health Center, Farmington, CT 06030, USA; ^3^Hartford Healthcare, Hartford Hospital, 80 Seymour Street, Hartford, CT 06102-5037, USA; ^4^Department of Reconstructive Sciences, University of Connecticut Health Center, Farmington, CT 06030, USA

## Abstract

Evidence-based integrative medicine therapies have been introduced to promote wellness and offset side-effects from cancer treatment. Energy medicine is an integrative medicine technique using the human biofield to promote well-being. The biofield therapy chosen for study was Therapeutic Touch (TT). Breast cancer tumors were initiated in mice by injection of metastatic 66cl4 mammary carcinoma cells. The control group received only vehicle. TT or mock treatments were performed twice a week for 10 minutes. Two experienced TT practitioners alternated treatments. At 26 days, metastasis to popliteal lymph nodes was determined by clonogenic assay. Changes in immune function were measured by analysis of serum cytokines and by fluorescent activated cells sorting (FACS) of immune cells from the spleen and lymph nodes. No significant differences were found in body weight gain or tumor size. Metastasis was significantly reduced in the TT-treated mice compared to mock-treated mice. Cancer significantly elevated eleven cytokines. TT significantly reduced IL-1-a, MIG, IL-1b, and MIP-2 to control/vehicle levels. FACS demonstrated that TT significantly reduced specific splenic lymphocyte subsets and macrophages were significantly elevated with cancer. Human biofield therapy had no significant effect on primary tumor but produced significant effects on metastasis and immune responses in a mouse breast cancer model.

## 1. Introduction

In the most comprehensive study to-date on the use of complementary and alternative medicine (CAM) therapies, approximately 40% of the U.S. population has used some type of CAM in 2007 with the most common being the use of natural products that were not minerals or vitamins [1]. Many Americans seek CAM therapies for their personal health and well-being. Therefore it is important to determine efficacy of particular integrative medicine/complementary therapies, especially since the majority of cancer patients have been shown to use these therapies [2–4]. Although energy medicine or human biofield therapies are a small part of integrative medicine, a recent study on cancer patients demonstrated that these patients reported the highest benefit with energy medicine compared to any other CAM therapies (*p* < 0.004) [3]. Scientific evidence for the possible reasons for this benefit is needed. As a first step we undertook a cancer study in animals to remove psychosocial factors.

The concept of a human biofield has its origins in many different cultures over thousands of years with the development of numerous types of biofield therapies: Reiki, External Qi Therapy, Healing Touch, Therapeutic Touch, and so forth, but only recently has Western science begun to evaluate these practices for their possible therapeutic potential. The purpose of many of these practices is to promote health, relaxation, and well-being. Therapeutic Touch was chosen for our study because it has one of the strongest histories of clinical trials demonstrating decreased anxiety in various clinical settings [5–7], decreased pain [8–11], diminished anxiety and pain [12, 13], improved functional ability in patients with arthritis [9, 14], decreased behavioral symptoms associated with dementia [15, 16], and enhanced personal well-being in persons with cancer [17]. Many studies are underpowered. However, in a comprehensive systematic review of 66 clinical trials, biofield therapies demonstrated strong and significant evidence for reducing pain and anxiety, and other palliative effects [18]. An earlier Cochrane review [19] that examined randomized controlled trials of biofield therapies for pain reported that biofield therapies improved pain compared to sham/mock treatment and no-treatment controls. A more recent systematic review of human randomized controlled trials of nontouch therapies for health-promoting effects identified 18 studies that met rigorous inclusion criteria, of which the majority was TT-based studies, and all studies found at least one significant beneficial outcome [20].

The mechanism by which human biofield therapies cause changes in living organisms is not entirely known. Rubik [21] defined the human biofield as an “endogenous, complex dynamic electromagnetic field” comprised of numerous electromagnetic fields or waves of different frequencies, which are capable of “self-organization and bioregulation of the organism.” In biology, the electromagnetic fields emitted by the heart and brain and other organs are well-accepted. Medicine and science measure the pattern of these electromagnetic biofields to monitor the health of the heart and brain through electrocardiograms (ECG), electroencephalograms (EEG), and magnetoencephalograms (MEG). Although the type of energy that may be produced by the human biofield is not known, electromagnetic fields may be a component of the mechanism by which the human biofield affects cells and organisms. Pulsed electromagnetic fields (EMFs) have been shown to inhibit tumor growth and tumor angiogenesis in animals [22, 23]. In a breast cancer model in mice, EMFs significantly reduced tumor growth and the extent of vascularization with increased tumor necrosis in animals [22, 24]. There have also been reports that continuous exposure to EMF can enhance the growth rates of transformed cells in culture for some human epithelial cancers [24]. In human biofield studies, very low electromagnetic fields have been detected from the hands of practitioners [25–27]. In another study, forces outside of the electromagnetic spectrum were shown to be a component of the human biofield [28]. Electromagnetic fields may play a role in the human biofield; however, other elements of the human biofield have not yet been identified.

Several reasons for choosing Therapeutic Touch for this study were the method of practice, which is an uncomplicated, well-defined protocol consisting of four steps, easily amenable for reproducibility of practice in a research trial and simple to perform in any setting [29]. The rigorous training program and credentialing process for practitioners, mostly nurses in all of our studies, was also important for consistency. There are no religious ties to the practice, so issues such as the role of prayer or religion are not involved in the interpretation of results. The first step in the practice is to set an intention, which is for the “highest good” of the subject. Finally, TT treatments do not require physical touch, so there is no heat transfer or variable handling of the subject being studied.

A breast cancer preclinical model was chosen for the study since it is the most common cancer in women in both developing and developed countries [30]. According to 2009–2011 data, approximately 12.3% of women will be diagnosed with breast cancer in their lifetime. Emerging evidence suggests that the tumor cell and multiple cellular elements in the microenvironment interact to promote carcinogenesis [31]. Bidirectional paracrine factors affect tumorigenic cell populations, which produce cytokines and growth factors that attract and regulate multiple cell types in the tumor. Cancer metastatic processes initiate with the transformation of the primary tumor cells into a phenotype that promotes unregulated growth, angiogenesis, breakdown of the extracellular matrix, intravasation, entry of metastatic cells into the circulation, cell adhesion to the endothelium of target organs, extravasation, and subsequent growth in new organs. To capture some of these processes we used a well-accepted but simple model of mouse breast cancer that has the ability to metastasize to the lymph nodes. The 66cl4 cell line is derived from an aggressive 4T1 mouse mammary carcinoma [32].

It is now accepted that the immune system has a causal role in breast cancer [33–35]; therefore, we determined changes in immune cells, as well as serum cytokine/chemokines in this cancer model. Since this is the first human biofield study to analyze a wide array of immune cells and cytokines, we chose a broad spectrum of lymphocytes and macrophages for fluorescent-activated cell sorting (FACS) to identify major changes in any particular class of immune cells that can then be studied in more detail in the future for their specific fingerprint.

## 2. Methods

### 2.1. Cells

The 6-thioguanine-resistant 66cl4 cell line was derived from an aggressive 4T1 mouse mammary carcinoma that can metastasize from the primary tumor to popliteal lymph nodes [32]. Metastasis can be quantified by counting cancer cell colony formation of dissociated lymph nodes (described below). Approximately 2 weeks prior to cell injection, cells were cultured from frozen stocks. Dr. Liisa Kuhn had these cells available at our institution and she received them from Dr. Miller, Karmanos Cancer Institute, Wayne State University, Detroit, Michigan [32].

### 2.2. Mice

Six–eight-week old, female BALB/c mice (Charles River, NCI) were received one week before study onset. All experiments were approved by the Animal Care Committee of the University of Connecticut Health Center. Twenty *μ*L of a 3.6 × 10^7^ cells/mL suspension in phosphate-buffered saline (PBS) was injected into the right rear footpad of all mice on the same day. Four-five mice were housed/cage. In the first study each treatment group consisted of 16 mice. An additional 8 mice received 20 *μ*L vehicle injections (PBS) as the negative control. For the second study, 12 mice/treatment group were used and an additional 8 mice for the negative control. Mice were randomly assigned to groups. At approximately 4 weeks, all mice had large tumors that began to interfere with ambulation, so mice were euthanized. All mice were weighed at the start and end. Euthanasia was performed on the same day for each experiment. Spleen, popliteal lymph nodes, and blood were immediately isolated.

### 2.3. Treatments

TT treatments commenced 24 h after cell injection and were repeated twice a week for the entire period. Two mice at a time were placed into large tissue culture flasks (Sarstedt, Newtown, NC, 18 cm × 11.5 cm × 4 cm) with bedding by a technician through a premade hinged door. Previous studies from our laboratory had shown that tissue culture plastic did not impede human biofield treatments [36]. Flasks were clamped two feet in the air in a ring stand at the end of an L-shaped room. Practitioners alternated treatments so that each practitioner treated mice once a week. Treatment lasted 10 min with hands kept 2–10 inches from all sides of the flask without touching (TT1). Briefly the treatment sequelae were centering, assessment, treatment, and evaluation and followed previously published protocols [29, 36]. The control/mock group consisted of placing two mice in a similar flask and setup for ten minutes twice a week (CA1) at the other end of the same L-shaped room with a non-TT person standing next to the flask. The third group of mice was PBS-injected and received no treatment (PBS1). On the 26th day, mice had developed large tumors in their foot pad and were euthanized.

After completion of the first study, a second study was undertaken in which mice were treated an additional two weeks by the TT practitioners prior to cell injection and throughout the study (TT2). The same protocol was followed as described. Mice were weighed and received a 20 *μ*L injection of cancer cells (3.7 × 10^7^ 66cl4 cells/mL) into their right rear footpad. The control/mock group of mice (C2) was also placed into flasks for 10 minutes twice a week for two weeks and then was injected with 66cl4 cells. Another group of mice was PBS-injected (PBS2). Twenty-four hours after cell injections, the protocols for TT1, C1, and PBS1 groups of mice were followed, as described for the first study. On the 29th day mice were weighed and euthanized.

### 2.4. Tumor Volume Measurements

Length and width of tumors were measured with a digital caliper. The size of the tumor was determined by the formula: tumor volume = (1/2)(length × width^2^) [37].

### 2.5. Flow Cytometry

From each group of 8 mice, spleens and popliteal lymph nodes were harvested, mechanically disrupted, and passed through a 70 *μ*m cell strainer (BD, Bedford, MA) to produce a single-cell suspension. Splenic erythrocytes were lysed by rinsing cells with deionized H_2_O. Lysis was terminated with Hank's Balanced Salt Solution (Sigma). Both popliteal and splenic cells were washed in FACS buffer (PBS, 0.2% bovine serum albumin (BSA) and 0.1% NaN_3_) and 10^6^ cells-aliquots were incubated with 100 *μ*L of appropriately diluted antibodies for 30 min at 4°C. The following monoclonal antibodies were used for mouse cell surface staining: CD44 (eFluor 450), CD49b (FITC), CD4 (APC), CD8a (PerCP-eFluor 710), CD11b (PE-Cy7), CD 19 (APC-eFluor 780), and CD25 (PE) (eBioscience, San Diego, CA). Cells were fixed with 4% paraformaldehyde and rinsed. Relative fluorescence intensities were determined on a 4-decade log scale by flow cytometric analysis, using an LSRII (Becton Dickinson, San Jose, CA). Five hundred thousand cell events were collected/sample. Analysis was carried out with FACSDiva software (BD Biosciences, San Jose, CA) by the University of Connecticut FACS facility without knowledge of sample identity.

### 2.6. Serum Cytokine Assay

Blood was collected via cardiac puncture, clotted, and centrifuged. Serum aliquots were frozen at −80°C until assayed. Cytokine levels were determined with the Milliplex Mouse Cytokine/Chemokine magnetic bead premixed 32-plex kit (MCYTMAG-70K-PX32, Millipore Co., Billerica, MA) according to manufacturer's protocol by the General Clinical Research Center at the University of Connecticut without knowledge of sample identity.

### 2.7. Metastasis Assay

Popliteal nodes on the tumor and nontumor sides were collected from 8 mice from mock and TT-treated groups. The nodes on the nontumor side served as negative controls. Each node was placed into one well of a 6-well plate with 3 mL of culture medium (60 *μ*m 6-thioguanine in RPMI-1640, 1 mM nonessential amino acids, 2 mM L-glutamine, 100 units/mL penicillin-streptomycin, 1% pyruvate, and 10% FBS). Each node was mechanically dissociated and incubated for 12 days in a cell incubator. The cancer cells are 6-thioguanine-resistant and are the only cells that survive. Cells were fixed with methanol, stained with 0.03% w/v methylene blue, rinsed, and dried. Without knowledge of treatment groups, the number of colonies/well was counted in the light microscope by two independent individuals.

### 2.8. Histology and Immunocytochemistry

Tumors from 4 mice/group were dissected, fixed in 4% paraformaldehyde, dehydrated, and embedded in paraffin. Approximately 5 *μ* sections were cut, deparaffinized in xylene, and rehydrated through graded alcohol steps. The APO-BRDU-IHC kit for measuring apoptosis by dual color immunohistochemistry was used according to manufacturer's instructions (Chemicon Int., Merck KGaA, Darmstadt, Germany). For determining proliferation, a polyclonal rabbit IgG against Proliferating Cell Nuclear Antigen (PCNA) was used (PA5-27214, Thermo Scientific, Rockford, IL) and a second antibody with an avidin-biotin complex (Vectastain Elite ABC Kit, Vector Laboratories) was used. For quantification, 4 mice/group and one section/mouse without tears or defects were chosen for analysis. The number of stained cells/field in 5 neighboring fields was quantified.

### 2.9. Statistics

Results were obtained from 8 mice/group/assay for the first study and 6 mice/group/assay for the second study as means ± standard error. Statistical comparisons were carried out by nonparametric ANOVA (Kruskal Wallis) followed by Bonferroni posttest with *p* < 0.05 considered to be statistically significant.

## 3. Results

Two weeks after 666c14 cell injection, tumors became evident in all mice. Due to rapid growth of tumors and necrosis, mice were euthanized at 26 and 29 days in the first and second studies, respectively. Visual inspection of major organs in each mouse revealed no additional tumor masses. Mice in all groups survived and gained weight with no significant differences in weight gain or tumor volume between groups (Table 1).

**Figure 1:**
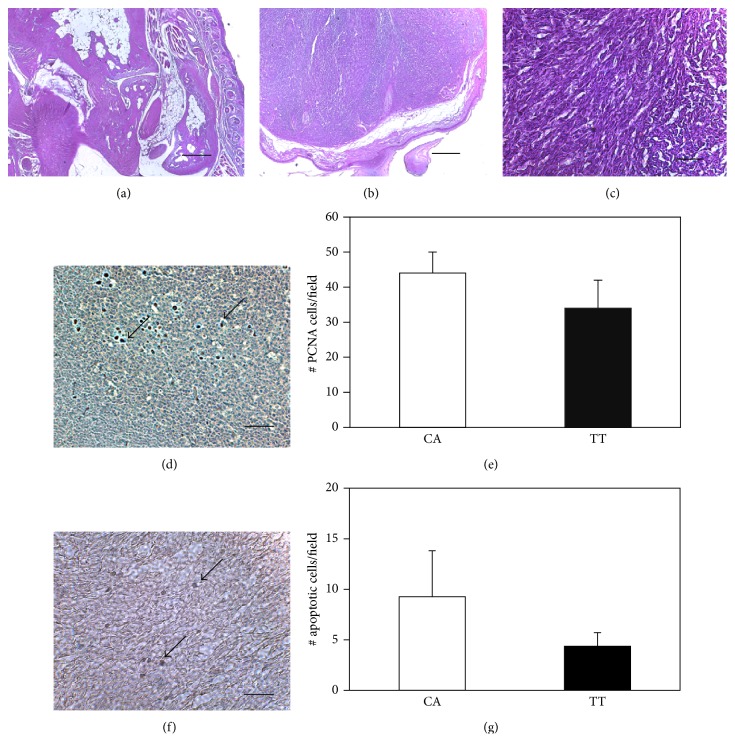
Proliferation and apoptosis in the primary tumor. In (a) the normal morphology of the foot is seen in a PBS-treated mouse (PBS). Panels (b) and (c) demonstrate at successively higher magnifications the change in the foot injected with 66cl4 cells from a mouse breast carcinoma (CA group). Mostly undifferentiated tumor tissue is found. In panel (d) PCNA-stained cells are seen in the TT-treated tumor (arrows), and the graph (e) shows no significant differences in proliferation between CA and TT groups. Apoptosis was assessed by immunohistochemistry (arrows) of the TT-treated tumor (f) and quantified in the graph (g) showing no significant differences between mice from the CA and TT groups. Bar = 250 *μ*m in (a) and (b). Bar = 50 *μ*m in (c), (d), and (f).

Figure 1(a) shows the histology of the normal mouse foot from a representative PBS-injected mouse. Foot pad tumor histology (Figures 1(b) and 1(c)) contain mostly undifferentiated tissue. PCNA immunocytochemistry demonstrated numerous proliferating cells (Figure 1(d)) in the tumor tissue, but there were no significant differences in PCNA-stained cell numbers between the TT- and the mock-treated tumor (CA) (Figure 1(e)). There were fewer apoptotic cells (Figure 1(f)) than proliferating cells; however, in a similar manner, no significant differences in apoptotic cell numbers were found between the TT and CA (Figure 1(g)).

**Figure 2:**
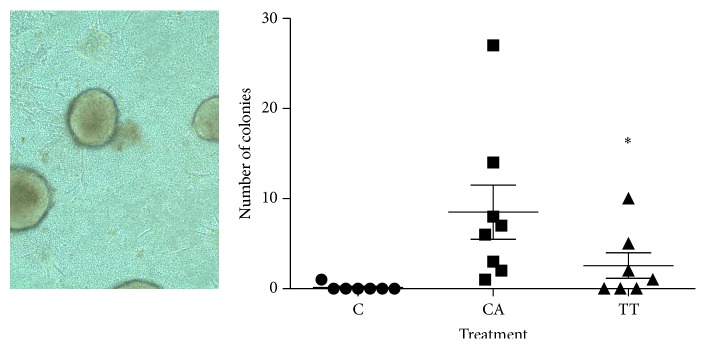
Effect of human biofield on clonogenic/metastasis assay. After 12 days in culture the total number of colonies from each dissociated lymph node was counted, and a representative field can be seen on the left. Large clonal masses of metastatic cells are found. On the left is a graph of the number of colonies/mouse. In the graph, the cultures of the lymph nodes from the uninjected contralateral limb demonstrated no colonies (C). The TT-treated mouse group (TT) had significantly fewer metastatic colonies compared to the mock-treated group (CA). In addition, all mice in the CA group had colonies while three mice in the TT group did not demonstrate any metastasis to the lymph nodes. Each point is from one mouse ± standard error of the mean of 8 mice. ^*∗*^ *p* < 0.05.

For the metastasis assay, most mice had 2–9 cancer cell colonies/lymph node. In the contralateral control limb (C), no tumors developed and no metastatic colonies were found (Figure 2). In contrast, every mouse had metastatic colonies in the mock-treated group (CA). In the TT-treated group (TT), three mice had no metastatic colonies while the remaining mice had some colonies. One mouse had 7-fold more colonies (76 colonies) than the mean. If this extreme outlier is excluded since it is greater than two standard deviations from the mean, TT significantly decreased metastasis compared to the mock-treated group (Figure 2).

Analysis of 32 serum cytokine/chemokine markers revealed an increase in 11 cytokines with cancer compared to PBS-injected mice: interferon gamma (IFN-*γ*), interleukin- (IL-) 2, IL-4, IL-5, IL-12 (p40), IL-1*β*, IL-1*α*, interferon gamma-induced protein-10 (IP-10), macrophage colony-stimulating factor (M-CSF), macrophage inflammatory protein-2-alpha (MIP-2), and monokine-induced by gamma interferon (MIG). Most of the remaining 21 cytokines were not detected. Some cytokines were detected but not significantly altered by treatments.

**Figure 3:**
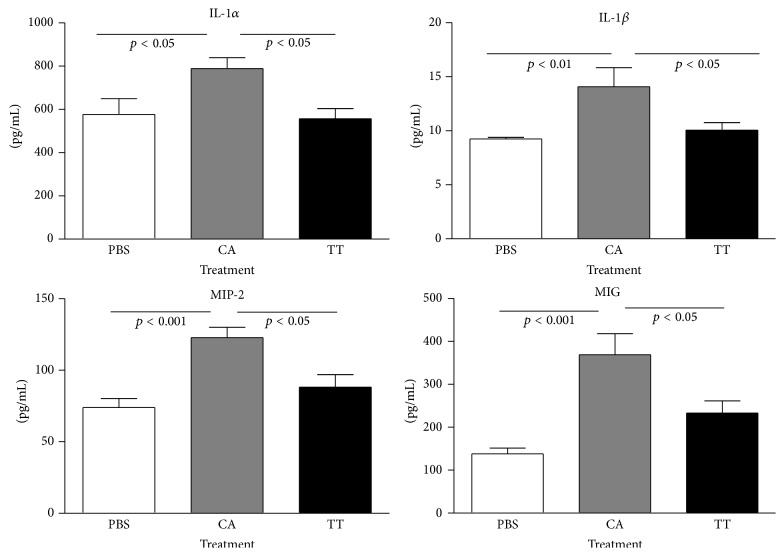
Effect of human biofield on serum cytokine/chemokine levels. Human biofield treatment (TT) significantly decreased IL-1*α*, IL-1*β*, MIG, and MIP-2 from their high levels in the mock treatment group (CA) to the levels found in the control group (PBS). Bars are means ± standard error of the means. *N* = 8 mice per group.

With human biofield treatment (TT), 4 of 11 cytokines significantly decreased to control levels compared to the serum from mice that were mock-treated (CA). IL-1*β*, IL-1*α*, MIP-2, and MIG were upregulated by cancer and downregulated to control levels (PBS-treated mice) by TT (Figure 3).

In the second study with TT pretreatment (TT2), changes in the same group of 32 cytokine/chemokine markers were analyzed. Of 11 markers previously affected, 7 markers significantly changed in the same manner: IL-1*β*, IL-1*α*, IFN-*γ*, MIP-2, IL-2, MIG, and IP-10, with cancer (CA2). Therefore, this breast cancer model had reproducible changes. Another 2 cytokines changed but instead of finding an increase in IL-5 and IL-12 (p40), they were significantly decreased with cancer (CA2) compared to their levels in the serum of the control mice (PBS2). No significant differences were found with IL-4 or M-CSF. Again in the second experiment, human biofield treatment (TT2) significantly decreased IL-1*β*, IL-1*α*, MIP-2, and MIG from high levels with cancer to control/PBS levels. Pretreatment of mice with TT did not enhance further the effects on the 4 cytokines but showed similar significant changes.

**Figure 4:**
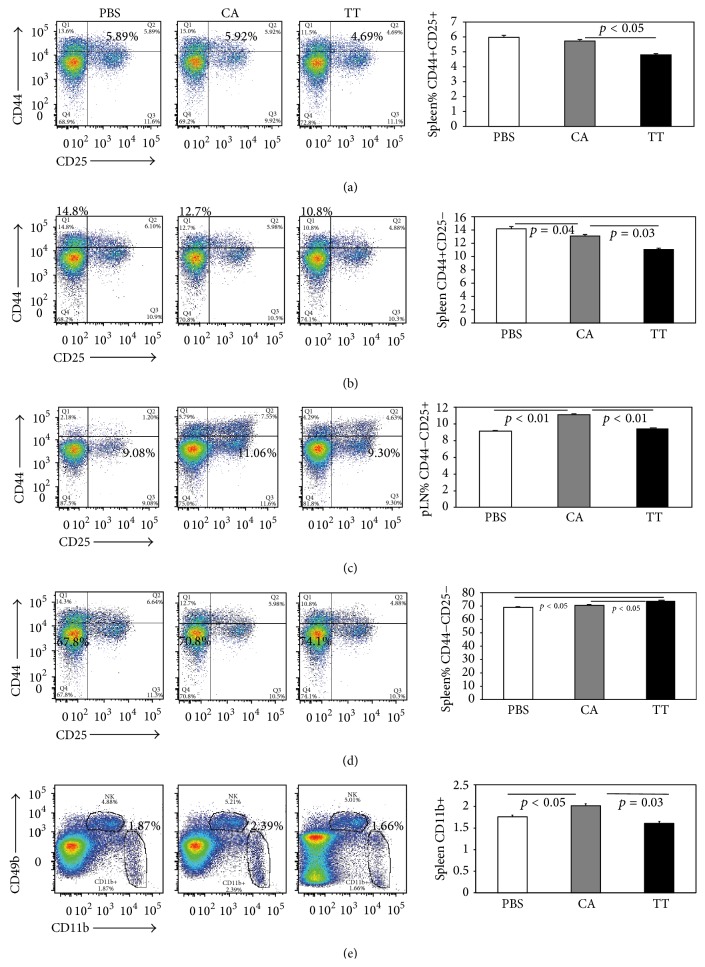
Flow cytometry scatter plots of immune cells from a representative mouse in each box (left) and graph (right) from the spleen or popliteal lymph node (pLN) of 8 mice with cancer treated with Therapeutic Touch (TT) compared to mice with cancer and mock treatments (CA) and mice that were PBS-injected without cancer (PBS). With TT treatment, % of CD44hiCD25+ were significantly decreased with TT treatment (TT) compared to PBS and CA (a). In (b) the % of CD44hiCD25− in the spleen was significantly decreased with TT (TT) compared to CA and the control (PBS). In (c) the % of CD44loCD25+ in the popliteal lymph nodes increased significantly with cancer (CA). With TT treatment (TT) these lymphocytes returned to levels comparable to those found in PBS-injected mice (PBS). In the spleen, TT treatment (TT) significantly increased the % of CD44loCD25− compared to CA and PBS (d). Finally, TT treatment (TT) significantly decreased the % of CD11b+ macrophages (TT) that were increased in the mock-treated cancer group (CA). Bars are means ± standard error of the means. *N* = 8 mice per group.

FACS analysis of specifically labeled macrophages and lymphocytes revealed significant changes with cancer and with TT. Graphs (Figure 4) illustrate the mean values of 8 mice in each of the 3 groups. A representative FACS plot of cells from a mouse closest to the mean is displayed next to each graph for each treatment group. The displayed gate frequencies are based on total lymphocytes. In the spleen the %CD4+ lymphocytes were not significantly different in any group. In the spleen, TT significantly decreased %CD4+CD44hiCD25+ lymphocytes compared to the mock-treated cancer group (CA) and control (PBS) (Figure 4(a)). CA and PBS groups were not significantly different from each other. Splenic %CD44hiCD25− lymphocytes were significantly decreased by TT compared to the control (PBS) and the mock-treated cancer group (CA) (Figure 4(b)). In the lymph nodes, cancer significantly increased %CD44loCD25+ lymphocytes (CA) versus the control group (PBS), and TT brought this level down to the control/PBS levels (Figure 4(c)). No other significant changes were found in the lymph node with TT treatment. In Figure 4(d), TT treatment (TT) significantly increased splenic %CD44loCD25− lymphocytes compared to control (PBS) and the mock-treated cancer groups (CA). In the spleen, macrophages, % CD11b+labeled cells were significantly increased in CA and were brought down to control levels (PBS) by TT (Figure 4(e)).

In the second study with TT pretreatment, practitioners did not elicit additional significant effects on labeled lymphocytes. Therefore, treatment with a human biofield had significant effects on serum cytokine/chemokine markers and immune cells once cancer began but had no additional effects if TT was given prior to cancer development. Thus, TT produced significant and reproducible decreases in immune cells elevated by cancer and brought them down to normal levels.

## 4. Discussion 

An aggressive mouse breast cancer model was created to study the effects of human biofield therapy. Therapeutic Touch (TT) treatment was able to significantly decrease metastasis but not primary tumor size compared to mock-treated mice with cancer. Two separate experiments demonstrated similar significant changes in 7 of 11 serum cytokine/chemokine markers in tumor-laden mice, suggesting that they have a role in this breast cancer model. TT produced significant effects in IL-1*α*, IL-1*β*, MIP-2, and MIG, which were upregulated with cancer and brought down to control (PBS) levels. Macrophages and some lymphocyte groups were also significantly decreased by TT, suggesting this human biofield therapy has significant effects on immune function, which may mediate the decrease in metastasis. However, pretreatment with TT prior to cell injection had no additional effect on metastasis, serum cytokines, or % immune cells.

The role of T-lymphocytes in breast cancer is paradoxical [34, 38, 39]. Although there is evidence that activated T-lymphocytes can destroy tumor cells* in situ*, they appear to be ineffective in destroying established cancer, and infiltrating T-lymphocytes can either eliminate or promote breast cancer development. Cancer research has now shown that a dysregulated immune system contributes to breast cancer progression. However, chronic inflammation is considered important for cancer progression by enhancing angiogenesis and tissue metastasis and releasing cytokines that promote carcinogenesis. Therefore, the downregulation of activated lymphocytes, CD44hiCD25+, CD44hiCD25−, and CD44loCD25+, suggests that TT may reduce metastasis through downregulation of these activated lymphocytes and additionally macrophages, and their respective cytokines. This novel finding is the first evidence that human biofield therapies may affect immune function in breast cancer.

Lymphocytes expressing CD44 and CD25 in the spleen were downregulated with an increase in CD44loCD25− lymphocytes, suggesting that TT treatment may reduce inflammation as seen by the decrease in circulating, activated T-lymphocytes induced by the mouse mammary carcinoma cells. Seemingly, TT was able to decrease the inflammatory response, which promotes tumorigenesis. This response probably plays a role in the decrease in metastasis with TT, but TT was unable to reduce the size of the primary tumor.

The 66cl4 cell-induced breast cancer model appears to be different from inflammation found in wound healing models since the cytokines modulated in this breast cancer model were different, even though the cancer cells were injected into the foot of the mouse and caused a large visible tumor. IL-6, MCP-1, and TNF*α* are elevated in mouse wound healing models [40, 41] but not in this breast cancer model, except for IL-1*β*, elevated in both models. Similar changes in cytokines were reproduced in separate experiments in this study demonstrating that TT-induced changes in immune function were related to breast cancer and not to wound healing.

TT significantly affected the % of macrophages and the levels of cytokines that activate macrophages and/or are produced by activated macrophages. These data suggests that TT specifically targets macrophages to elicit significant effects in breast cancer. Macrophages have been shown to promote tumor progression and metastasis in breast cancer [42, 43]. TT significantly decreased the % CD11b+ macrophages and brought down the levels of macrophage-related cytokines, IL-1*α*, IL-1*β*, and MIP-2, to normal levels. Interestingly, macrophage suppression by propranolol in stressed mice injected with 66cl4 cells was shown to inhibit metastasis, but not primary tumor growth, similar to our findings [44]. Chronic stress modulators from the sympathetic nervous system and the neuroendocrine system have been shown to have a profound effect on immune cells and the severity of disease in breast and ovarian cancer in humans [44, 45]; therefore, one of the mechanisms by which human biofield therapies may affect disease progression is to reduce stress.

A possible explanation of our findings is that the mice recognize and respond positively in a psychosocial manner to the biofield practitioner [46]. In studying psychosocial stress with inflammation and cancer, mouse models have shown that specific psychosocial stress factors produce generalized immune dysfunction, which particularly affects cytokine production resulting in changes in the numbers and function of specific leukocytes [47]. An alternative explanation of our findings is that the opposite of stress, such as exposure to a familiar and nonthreatening individual, may promote normal immune function. Mice attribute human contact with food, water, and positive environmental stimulation. Recently, rodents have been shown to detect and respond to the state of their social partners [48], and perhaps rodents may also respond positively to repeated human interactions. Thus, mammals may be capable of “felt affective experiences” [48]. On the other hand, mice that were placed in a similar apparatus by the same non-TT individual (CA group) did not manifest these changes in immune function suggesting that the TT treatment itself was responsible for the significant effects.

Our previous studies with human biofield therapies have shown significant effects on cancer cells* in vitro*. TT treatment of osteosarcoma cells, SaOs-2, significantly decreased mRNA levels of proteins involved in osteoblast differentiation and bone formation compared to mock-treated and untreated groups [29, 36]. Conversely, normal cells demonstrated increased proliferation and differentiation with TT treatment in a dose-dependent manner [49].

In this study, we found no significant effects of TT on proliferation or apoptosis in the primary tumor. This lack of effect may be due to differences in* in vivo* versus* in vitro* systems or the aggressiveness of the breast cancer model that is not responsive enough to TT to produce significant effects. This finding may be a limitation of this study since a less aggressive model of breast cancer may have more effects on the primary tumor with additional TT treatments or the primary tumor may be unresponsive to these types of therapies. This result also brings into question whether there is a dose response to human biofield therapies in animal models, which is yet to be studied. Perhaps the most fundamental challenge facing biofield researchers is the uncharacterized nature of the biofield itself, which makes determining experimental conditions difficult and subject to possible problems with reproducibility. However, changes in immune function with TT treatment were able to be reproduced twice in this study. Repetition of our findings from another independent laboratory, especially on metastasis, would also help to determine whether human biofield therapies are beneficial in cancer models. In this regard, an early study by Grad and colleagues reported that a biofield practitioner improved wound healing in mice, which confirms that mice are responsive to biofield therapies [50].

There have been few studies on the human biofield's ability to affect immune function with cancer. Lutgendorf et al. demonstrated that Healing Touch maintained NK cells cytotoxicity during radiation and chemotherapy in patients with cervical cancer, while relaxation and standard care groups of patients had a sharp decline in these important immune cells [51]. In their studies with 19–21 patients per group, the Healing Touch group received approximately fifteen 20-minute sessions. In our model of breast cancer, we found an upregulation of NK cells with cancer, which was further upregulated with human biofield therapy, but these data were not statistically significant with our small *N* and were not presented. In the future, it will be important to determine how the human biofield therapy affects immune function and disease outcome in cancer, both in patients and in preclinical models.

## 5. Conclusions

In a breast cancer model in mice, Therapeutic Touch had no significant effect on primary tumor size but significantly decreased metastasis. This human biofield therapy also significantly downregulated specific lymphocytes, macrophages, and serum cytokines induced by cancer. This study is the first to show modulation of immune function by human biofield therapies with possible positive outcomes in breast cancer progression. Human biofield therapy along with standard allopathic care may have beneficial effects on cancer subjects.

## Figures and Tables

**Table 1:** Changes in mouse weight and tumor volume. There were no significant differences in the weights between treatment groups either at the start (weight 1) or end (weight 2) of experiments. Mice injected with cancer cells (CA and TT) increased their weights in a similar manner as PBS-injected mice. Tumor volumes were not significantly different between CA and TT in either experiment.

Treatment group	Weight 1	Weight 2	Tumor volume
	(gm)	(gm)	(mm^3^)
Experiment 1			
PBS	18.1 ± 0.3	19.8 ± 0.2	0
CA	18.1 ± 0.2	19.4 ± 0.3	222.6 ± 18.1
TT	18.3 ± 0.1	20.1 ± 0.2	220.8 ± 18.5
Experiment 2			
PBS	20.3 ± 0.3	21.4 ± 0.4	0
CA	20.7 ± 0.3	21.4 ± 0.5	320.3 ± 30.6
TT	20.8 ± 0.2	21.1 ± 0.3	330.8 ± 42.3
